# Graphynes: an alternative lightweight solution for shock protection

**DOI:** 10.3762/bjnano.10.154

**Published:** 2019-07-31

**Authors:** Kang Xia, Haifei Zhan, Aimin Ji, Jianli Shao, Yuantong Gu, Zhiyong Li

**Affiliations:** 1 College of Mechanical & Electrical Engineering, Hohai University, Nanjing 210098, China; 2 School of Chemistry, Physics and Mechanical Engineering, Queensland University of Technology (QUT), Brisbane QLD 4001, Australia; 3 State Key Laboratory of Explosion Science and Technology, Beijing Institute of Technology, Beijing 100081, China

**Keywords:** graphyne, in silico studies, stress wave propagation, supersonic-velocity impact

## Abstract

The excellent mechanical properties of graphyne (GY) have made it an appealing candidate in the field of impact protection. We assessed the deformation mechanisms of monolayer GY nanosheets of different morphologies, including α-GY, β-GY, γ-GY and 6612-GY, under supersonic-velocity impacts (from 1 to 6 km/s) based on in silico studies. Generally, cracks initiate at the geometry center and the nanosheet experiences significant out-of-plane deformation before the propagation of cracks. Tracking the atomic von Mises stress distribution, it is found that its cumulative density function has a strong correlation with the magnitude of the Young’s modulus of the GYs. For nanosheets with a higher Young’s modulus, it tends to transfer momentum at a faster rate. Thus, a better energy dissipation or delocalization is expected during impact. This study provides a fundamental understanding of the deformation and penetration mechanisms of monolayer GY nanosheets under impact, which is crucial in order to facilitate their emerging applications for impact protection.

## Introduction

Owing to its versatile flexibility, carbon is able to form three different hybridization states namely sp, sp^2^ and sp^3^, which yields many kinds of carbon allotropes. Graphite and diamond are the two common carbon allotropes in nature. They consist of networks of sp^2^- and sp^3^-hybridized carbon atoms, respectively. Replacing a carbon–carbon bond in graphene by a acetylenic triple bond (–C≡C–), two-dimensional sp–sp^2^-hybridized GY is obtained [1]. Depending on the percentage of acetylenic linkages, different types of GYs have been reported, including α-GY, β-GY, γ-GY and 6612-GY [2].

Since the early 1990s, continuous efforts have been made to produce GY fragments with periodic lattice structure [3]. So far, homogenous sheets of γ-GY [4–5] and β-graphydine (a β-GY allotrope with longer acetylenic linkages) [6] have been fabricated experimentally utilizing cross-coupling methods and a modified Glaser–Hay coupling reaction, respectively. Regarding other GY allotropes, Yuan Q. et al. numerically illustrated the formation of GYs on transition metals via the self-assembly of carbyne chains [7]. Above efforts make the realization of entire monocrystalline GYs possible in foreseeable future. As an atomically thin 2D carbon nanostructure, its application in various fields is currently explored. For instance, the relatively large pore sizes in GY allows for a usage in desalination and water purification [8]. GY has a non-zero bandgap, which indicates a potential application in next-generation carbon-based semiconductors [9]. In addition, the properties of GYs are highly tunable through the modification of the topology. For instance, GYs are found to absorb light in the HOMO–LUMO band and the energy of the absorbed light is tunable by structural modifications of GY (e.g., through increasing the length of the GY chains) [10]. GYs can also be used as Li accommodator in battery anodes, which increases service life and safety of batteries. Under biaxial tensile strain, GY accommodates more Li particles, which reduces the diffusion barriers of Li in batteries [11].

The understanding of the mechanical performance of GYs is critical and fundamental for the implementation of GYs. Generally, GYs exhibit low density and great structure versatility with outstanding thermal and mechanical stability [12–14]. Based on in silico molecular dynamics (MD) tensile tests, the recorded failure strength values for different types of GYs range between 32.48 and 63.17 GPa [2,15–16]. According to a first-principle study, the failure strain of GY reaches 20% [17]. A high Young’s modulus of 532.5 GPa is reported for GYs using reactive force field (ReaxFF) potential calculations [15]. These results indicate excellent mechanical properties of GYs compared with conventional engineering materials [18–21]. Therefore, considering their low density, it is of great interest to investigate the application of GYs in the field of impact protection such as combat armor and protective shield against orbital debris for spacecraft [22]. Currently, no studies have investigated the performance of GYs under direct impact in literature. However ballistic tests have been conducted both experimentally and numerically on their counterpart graphene. Studies have revealed that carbon chains in graphene are surprisingly stable under electron bombardment [23–25]. Applying a miniaturized ballistic setup, the specific penetration energy of multilayer graphene has been reported to be more than 10 times higher than that of metal protection materials [26]. Based on this understanding, several in silico works followed. Axial-wave and cone-wave propagation patterns of graphene sheets under supersonic-velocity impact allows the graphene sheets to transfer more momentum per unit area and hence provide better ballistic protection [27]. Utilizing MD methods, a study suggests that the impact resistance of graphene is strongly correlated with the density of monovacancies [28]. Our recent work reveals the transmission of stress waves would eventually influence the penetration energy and crack growth in graphene [29]. This work will investigate the mechanical behavior of GYs with different acetylenic linkage percentages and morphologies under various impact loading for the first time. Their impact resistance performance will be compared accessing (specific) penetration energy, stress propagation, stress distribution performance as well as the number of broken bonds.

## Results and Discussion

**Figure 1:**
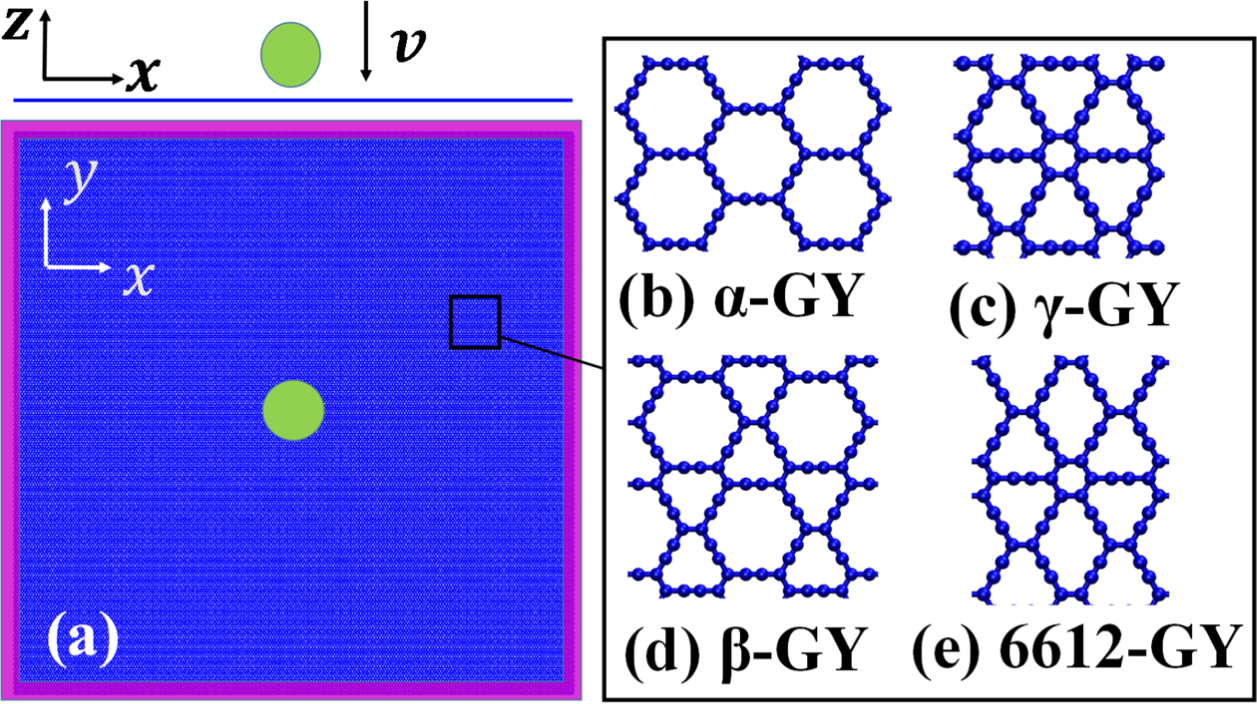
Schematic view of the impact simulation setup. (a) Top: side view and bottom: top view. The red regions represent the fixed boundaries during impact; atomic configurations of: (b) α-GY; (c) γ-GY; (d) β-GY; and (e) 6612-GY.

The fracture behavior and mechanical performance of GYs, including α-, β-, γ- and 6612-GY under supersonic-velocity impact were assessed through a series of large-scale MD simulations performed using the open-source package LAMMPS [30]. A spherical diamond projectile with a radius of 25 Å was adopted. A square monolayer GY nanosheet was constructed with a size of about 50 × 50 nm^2^. A high velocity of 20 Å/ps (i.e., 2 km/s) was chosen to initiate the impact test. The boundaries of GY were fixed during impact (Figure 1), and the sample has a zigzag edge in *x*-direction (armchair edge in *y*-direction). The projectile was located above the middle of the sample with an initial distance of ca. 8 Å.

### Deformation characteristics of α-GY under impact

**Figure 2:**
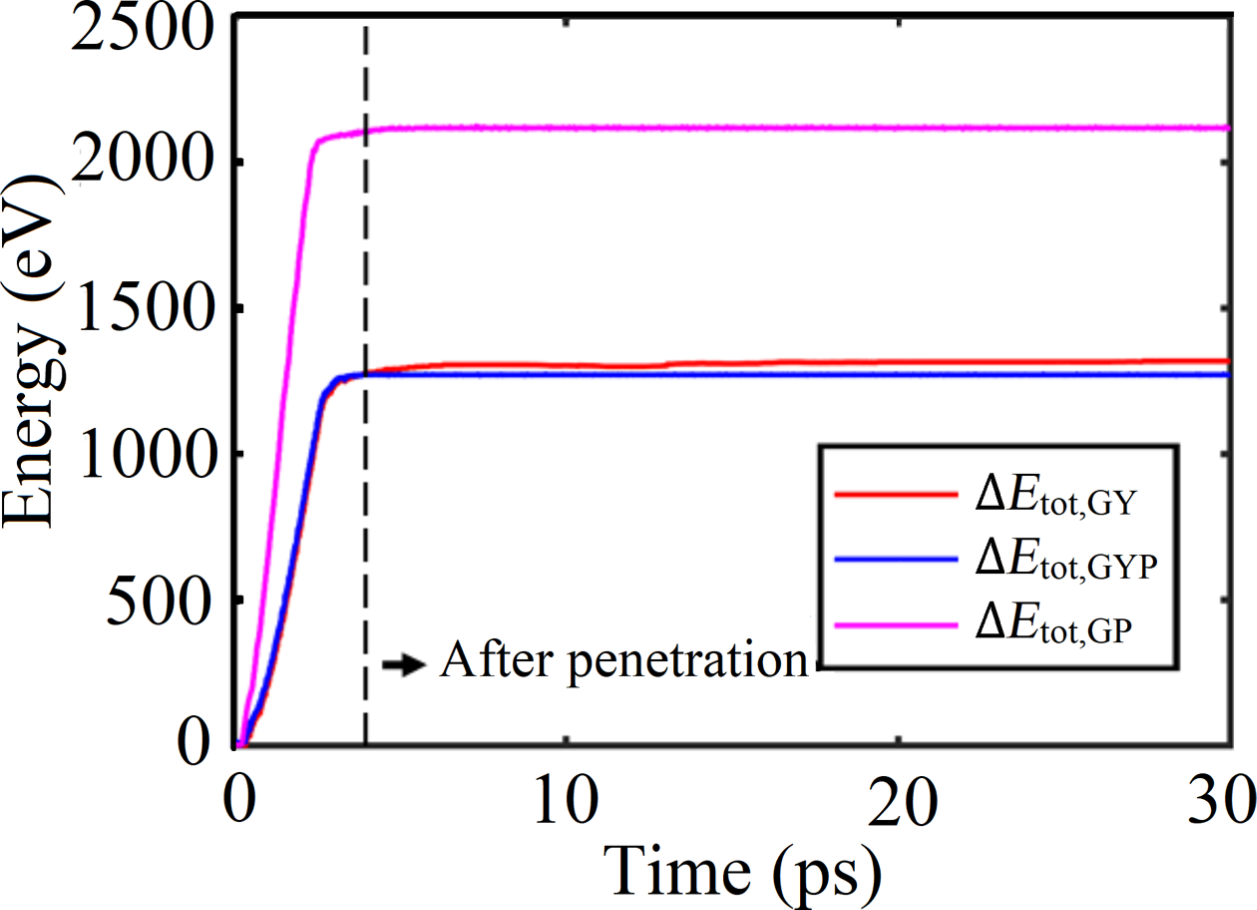
Energy change as a function of the time for an α-GY nanosheet under an impact velocity of 2 km/s. *∆E*_tot,GY_ and *∆E*_tot,GYP_ represent the total energy change of α-GY nanosheet and the projectile, respectively.

Initially, we focus on the impact performance of the α-GY nanosheet under an impact velocity of 2 km/s. The total energy change of the α-GY nanosheet (*∆E*_tot,GY_) and the corresponding projectile (*∆E*_tot,GYP_) is compared in Figure 2. Here the total energy *E*_tot_ of the system includes the kinetic energy and potential energy. Ideally, the change of the total energy of the projectile equals to that in the α-GY nanosheet. According to Figure 2, Δ*E*_tot,GYP_ nearly overlaps with Δ*E*_tot,GY_ before penetration. After perforation, *∆E*_tot,GYP_ remains constant because of the vacuum condition, whereas *∆E*_tot,GY_ increases slightly before saturating to a certain value. Such a variation of *∆E* is supposed to result from the fixed boundary conditions [29]. The total energy loss of the projectile after perforation is taken as the penetration energy (*E*_p_), which is about 1329 eV. According to a previous work, a graphene nanosheet (with a similar simulation setting) shows a penetration energy of around 2118 eV, which is larger than that of α-GY. However, considering the different atomic configurations, the values of the penetration energy per atom are very similar, i.e., ca. 0.020 eV and ca. 0.017 eV for α-GY and graphene nanosheets, respectively. During the impact, the projectile exhibits only negligible deformation (see Section 1 of Supporting Information File 1).

**Figure 3:**
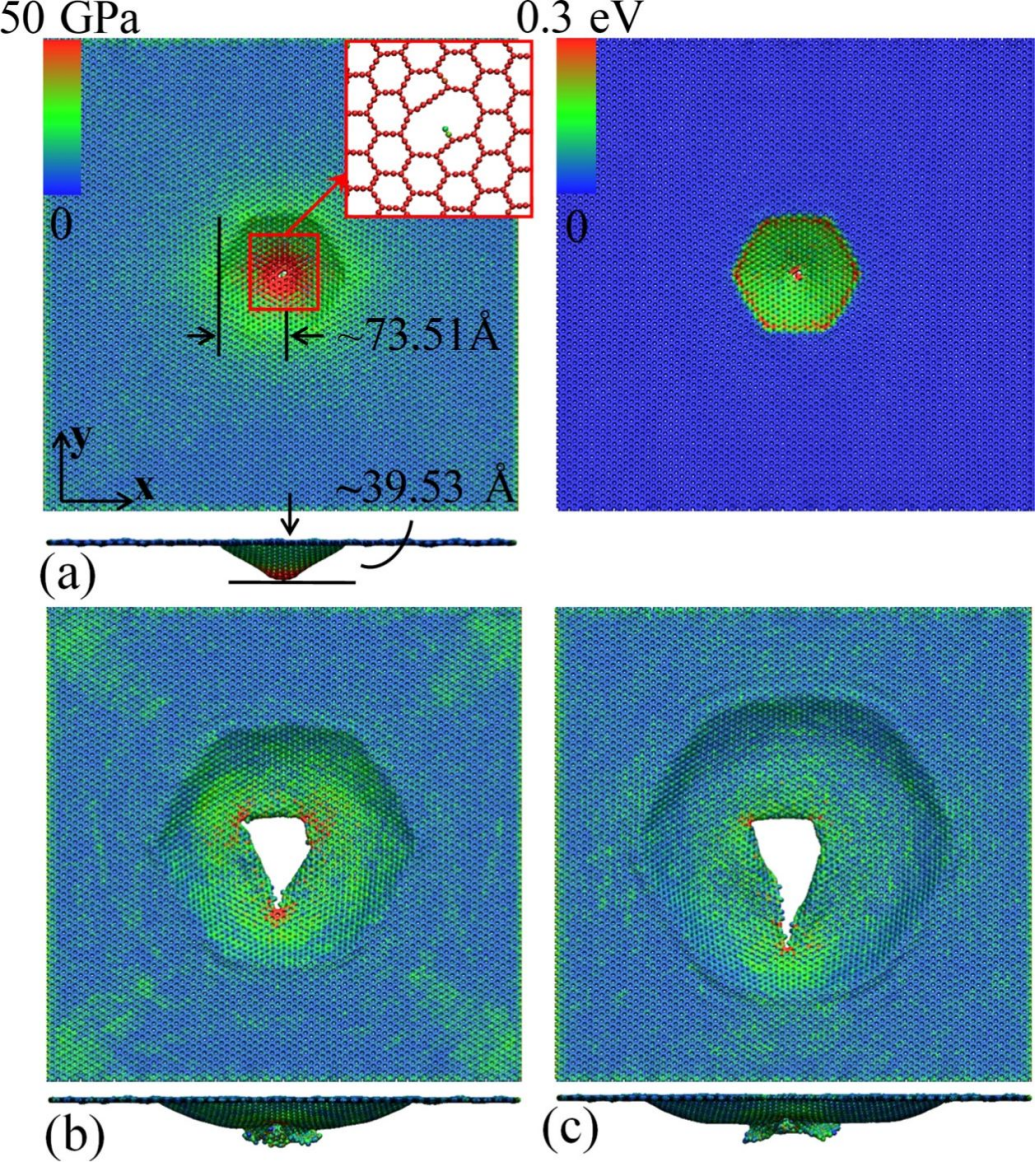
Impact deformation of α-GY under an impact velocity of 2 km/s. (a) von Mises atomic stress distribution pattern at a simulation time of 2.5 ps (left panel), and the corresponding kinetic energy distribution pattern (right panel). The insert in the left panel shows the formation of initial cracks at the impact area; (b) stress distribution pattern right after perforation at 5.0 ps; and (c) the final atomic configuration at 6.4 ps.

Figure 3 illustrates the atomic configurations of α-GY at different stages of the deformation process. As expected, a crack initiates at the geometry center and the nanosheet experiences significant out-of-plane deformation (ca. 39.53 Å) before the propagation of the crack (left hand side of Figure 3a). It is interesting to find that the kinetic energy (transferred from the projectile) in the deformed region exhibits a hexagonal pattern (right hand side of Figure 3a), which is considered a result from the relatively large hexagonal lattice structure of α-GY. During crack propagation, the initial stress that accumulated around the impact region starts to re-distribute, and stress concentrations at the crack tips are observed (Figure 3b). Different from the pure zigzag kicking fracture mechanisms in graphene [29,31], the cracks in α-GY propagate along both armchair and zigzag directions. After full perforation at ca. 6.4 ps, the crack propagation stops and the stress concentration at the tip is fully released (Figure 3c).

### Influence of morphology

It is of great interests to compare how the mechanical performance of GY would change with the variation of its morphology. In this regard, we apply the same simulation settings to other types of GY (with an impact velocity of 2 km/s), namely β-, γ- and 6612-GY. We found that β- and 6612-GY show smaller penetration energy values than α-GY (ca. 1200 eV and ca. 1066 eV, respectively), and γ-GY exhibits a slightly higher penetration energy (ca. 1368 eV, see Section 2 of Supporting Information File 1). The biggest difference is found between γ-GY and 6612-GY, which is around 300 eV. Such a big difference suggests a profound impact from the morphology of the GY on the impact performance.

**Figure 4:**
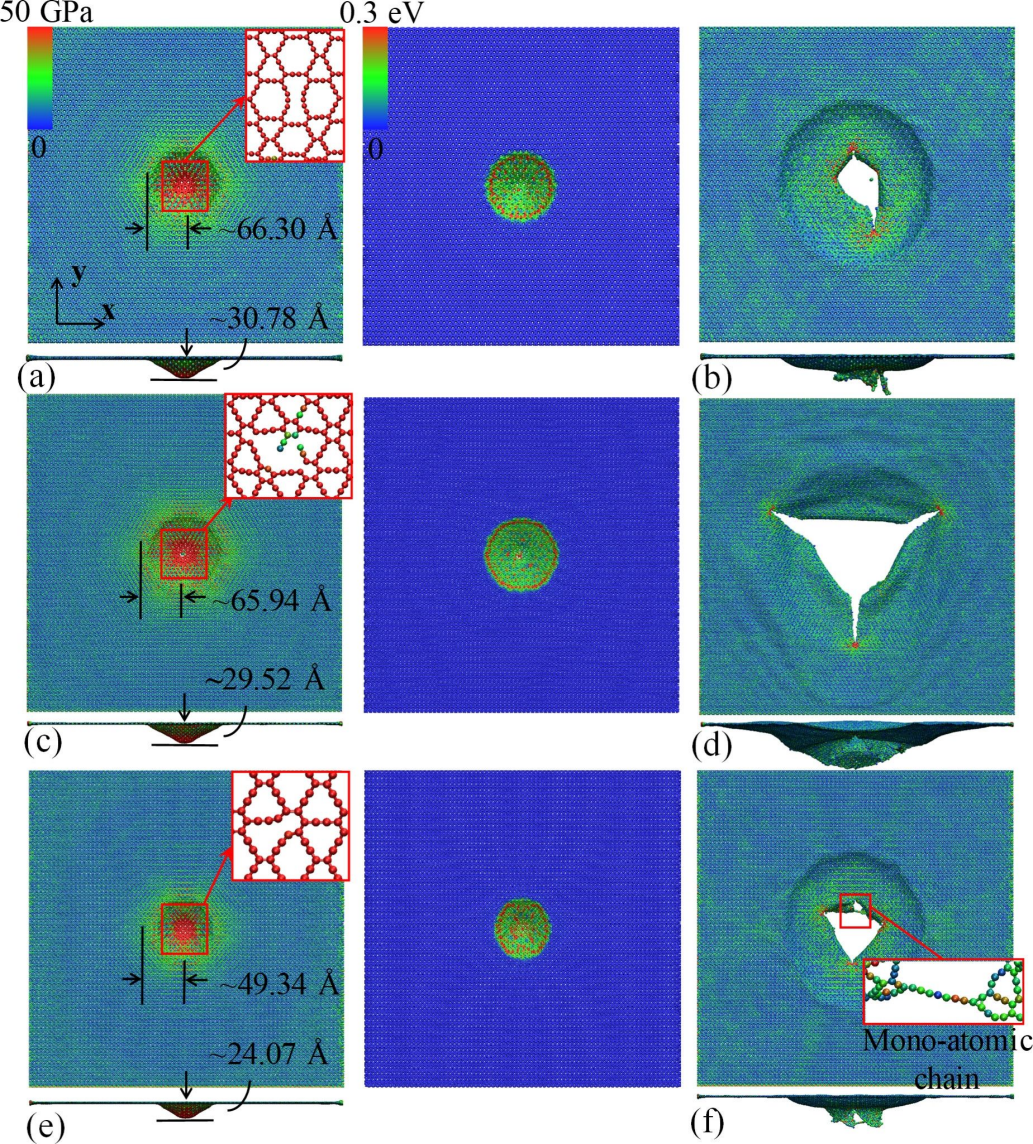
Impact deformation of different GYs under an impact velocity of 2 km/s. (a, b) Atomic configurations of β-GY: (a) von Mises atomic stress distribution pattern at a simulation time of 2.0 ps (left panel), and the corresponding kinetic energy distribution pattern (right panel); the insert in the left panel shows the formation of initial cracks at the impact area; and (b) final atomic configuration after 4.9 ps. (c, d) Atomic configurations of γ-GY: (c) von Mises atomic stress distribution pattern at a simulation time of 20 ps (left panel), and the corresponding kinetic energy distribution pattern (right panel); the insert in the left panel shows the formation of initial cracks at the impact area; and (d) final atomic configuration after 9.7 ps. (e, f) Atomic configurations of 6612-GY: (e) von Mises atomic stress distribution pattern at a simulation time of 1.6 ps (left panel), and the corresponding kinetic energy distribution pattern (right panel); the insert in the left panel shows the formation of initial cracks at the impact area; and (f) final atomic configuration after 4.5 ps.

In line with the different energy absorption capability, the GYs exhibit different deformation mechanisms. For β-GY, the out-of-plane deformation is about 30.78 Å before crack initiation. Instead of the hexagonal deformation pattern observed in α-GY (left panel in Figure 3a), β-GY shows a clearly round kinetic energy transmission region (right panel in Figure 4a). The cracks stop propagation at about 4.9 ps, which is much earlier than in α-GY (Figure 4b). In comparison, γ-GY shows a smaller out-of-plane deformation (ca. 29.52 Å) and a round kinetic energy transmission region similar to that of β-GY (Figure 4c). Three initial cracks are observed that continue to grow after full perforation and stop propagation at ca. 9.7 ps. These relatively large cracks induce three dangling petals after perforation (Figure 4d), which also explains the larger penetration energy compared to its counterparts. For 6612-GY, an earlier bond breakage is observed, which results in a small out-of-plane deformation (ca. 29.87 Å, left panel of Figure 4e). It is interesting to note that the kinetic energy transmission region exhibits an elliptical shape (right panel of Figure 4e), which is expected because of the anisotropic mechanical properties, which will be discussed later. Specifically, a monoatomic chain is formed during the deformation, which prevents the further propagation of the corresponding crack (Figure 4f).

**Figure 5:**
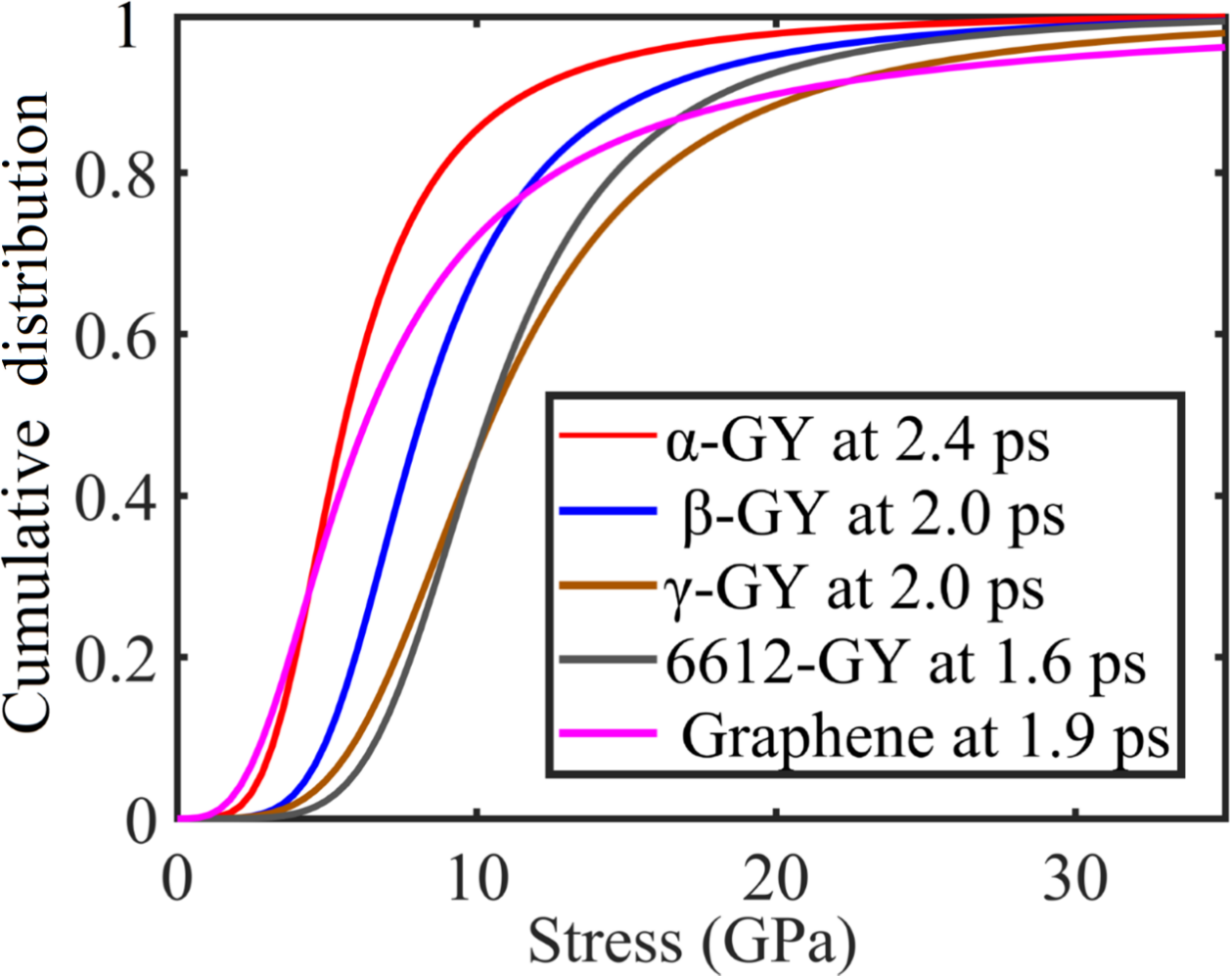
Cumulative distribution function (CDF) of the von Mises atomic stress distribution before crack initiation for different nanosheets under an impact velocity of 2 km/s.

To unveil the mechanisms that lead to the different performance of GYs under impact, the cumulative distribution function (CDF) of the von Mises atomic stress in each GY before the initiation of the crack is analyzed. According to Figure 5, the profile of CDF shows a strong correlation with the magnitude of the Young’s modulus (see Section 3 of Supporting Information File 1). In detail, α-GY has the smallest Young’s modulus and the CDF of its atomic stress converges to the value of 1 faster than all other curves, followed by and β-GY, 6612-GY, γ-GY and graphene. It is noted that γ-GY exhibits the highest Young’s modulus among the GYs, indicating the widest stress spread and most evenly distributed stress across the nanosheet prior to bond breakage.

Since the stress distribution is largely determined by the propagation of elastic stress, it is of great importance to compare the stress wave propagation velocity in the examined GYs. In a GY with higher elastic wave propagation velocity, the momentum tends to be transferred at a faster rate, thus, a better energy dissipation or delocalization is expected during impact. In this regard, we track the location of the highest stress during the simulation and estimate the elastic stress wave velocity (perpendicular to the fixed boundaries). As listed in Table 1, graphene exhibits the highest propagation velocities, which are 19.84 km/s and 20.11 km/s in *x-* and *y*-direction, respectively. It is followed by γ-GY with values of around 17.9 km/s in both *x-* and *y*-direction. Here, *x-* and *y*-axis are the lattice directions shown in Figure 1, which are the armchair and zigzag lattice directions, respectively, for graphene and α-GY. Among all examined nanosheets, α-GY shows the lowest elastic wave propagation velocity. In theory, the elastic stress wave velocity *v*_s_ in a solid material can be calculated from its Young’s modulus *E* and density ρ:


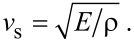


Based on the reported Young’s modulus values in literature [2,32] and the calculated density based on mass *m* and volume *V* of our samples (ρ = *m*/*V*), Table 1 shows a good agreement among the simulation results and the theoretical predictions, except for α-GY. Interestingly, γ-GY shows the best isotropic properties with similar values of ca. 17.9 km/s along both *x*- and *y*- direction. In contrast, a notable propagation velocity difference was found in 6612-GY, which is also well reflected by the elliptical stress wave shape in Figure 4e.

**Table 1:** Elastic stress wave propagation velocity (km/s) of different GYs and graphene. *x-* and *y*-axis are the lattice direction shown in Figure 1. For graphene and α-GY, *x-* and *y*-axis are the zigzag and armchair lattice directions, respectively. The values in brackets are from theoretical calculations based on the reported Young’s modulus from literature [2,32].

lattice direction	graphene	α-GY	β-GY	γ-GY	6612-GY

*x*-axis	19.84 (21.27)	14.49 (10.46)	15.61 (14.24)	17.9 (18.01)	17.65 (17.30)
*y*-axis	20.11 (21.28)	15.76 (10.41)	16.34 (14.21)	17.9 (18.06)	14.71 (15.35)

### Deformation under different impact velocity

Above discussions shown that the morphology will alter the deformation mechanism of GY under impact. Considering the different elastic stress wave propagation velocities, it is crucial to examine how the GYs will performance under different impact velocity amplitudes. For this purpose, we consider the deformation of an α-GY nanosheet under impact velocity values ranging from 1 to 6 km/s. It is found that to perforate the examined α-GY nanosheet, the velocity of the projectile should be larger than a threshold value of ca. 1.6 km/s. For impact velocity values lower than this threshold value, the projectile will be bounced back by the nanosheet. Similarly, the threshold velocity for β-GY, γ-GY and 6612-GY are 1.8 km/s, 1.8 km/s, and 1.6 km/s, respectively. Higher impact velocities will induce more severe local deformation, and there will be no time for the stress to develop a well distributed pattern as observed scenarios with in lower impact velocity. Extensive elastic deformation of the nanosheet will not occur. According to the atomic configurations (see Section 4 of Supporting Information File 1), the contact region melts immediately when the projectile approaches the GY with high impact velocity, which creates lots of dangling bonds. This phenomenon is uniformly observed in the examined GYs.

**Figure 6:**
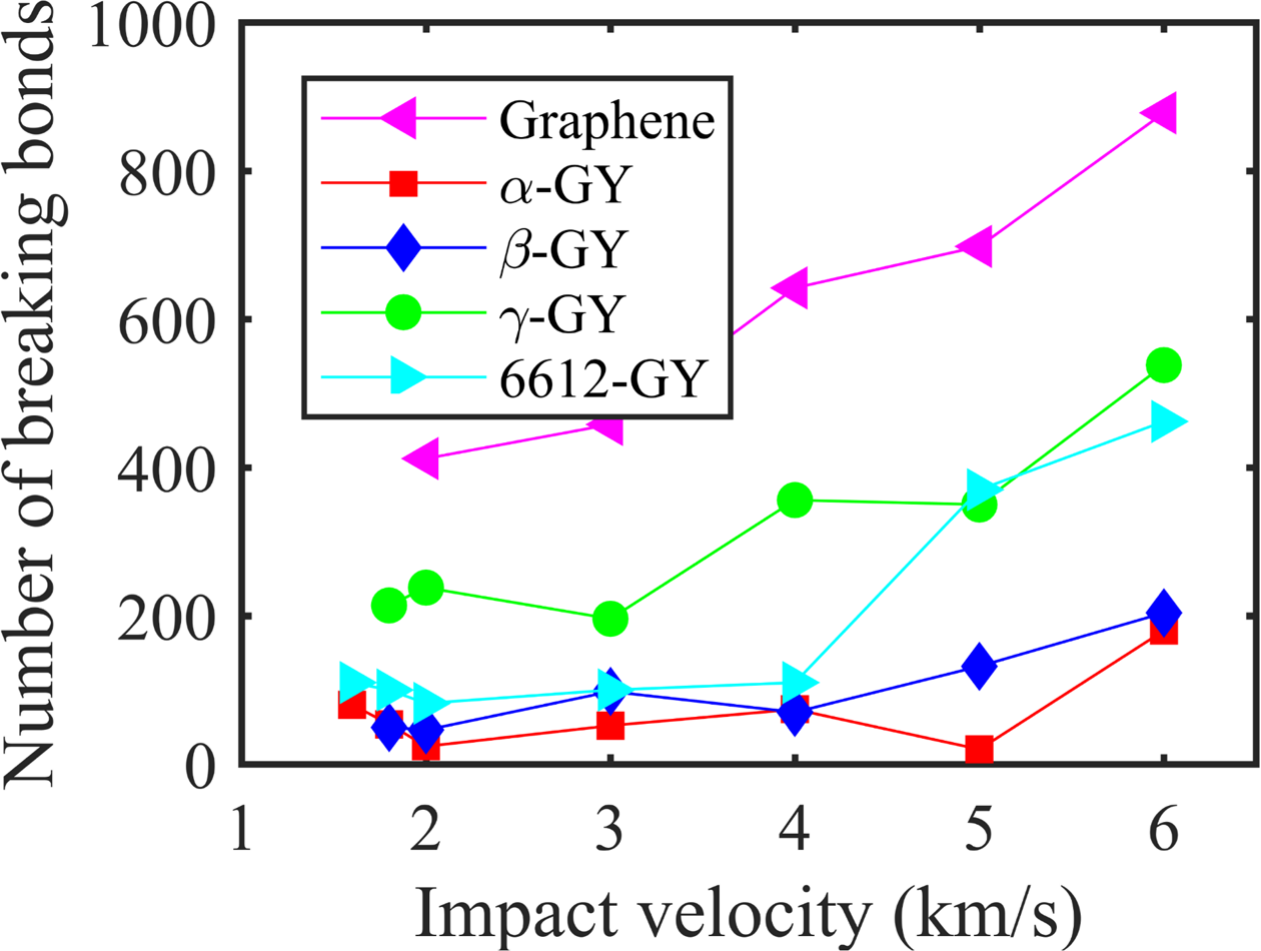
Number of breaking bonds in GY and graphene nanosheets under different impact velocity amplitudes.

To assess the performance of GYs under different impact velocity amplitudes, we track the number of breaking bonds by comparing the total number of bonds in the nanosheet before impact and after perforation (when all cracks stop to propagate). Figure 6 shows that the number of breaking bonds has a generally increasing tendency with the increase of impact velocity. Among all tested samples, α-GY and β-GY present the lowest number of breaking bonds at the examined impact velocity range. Although 6612-GY shares a similar number of breaking bonds at lower impact velocity with α- and β-GY, the number increases significantly to ca. 400 when the impact velocity reaches 5 km/s. In comparison, γ-GY and graphene show a much higher number of breaking bonds. It is worthy to note that the structures with a higher percentage of acetylenic linkages tends to have a lower number of breaking bonds, which means the presence of acetylenic linkages makes the material less brittle. Additionally, we conducted additional simulations by varying the time step from 0.1 to 0.5 fs from which the same results are obtained (see Section S5 of Supporting Information File 1).

To quantitatively compare the impact resistance of GY, we compare the penetration energy for each examined sample under different velocity amplitudes. Theoretically, the penetration energy can be estimated from


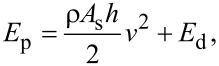


when the projectile diameter *D* is much larger than the thickness *h*, i.e., *D*/*h* ≫ 1 [26]. Here, the first term refers to the minimum inelastic energy transferred to the sample and the second term represents the contribution from other energy dissipation mechanisms, e.g., bond breakage. *A*_s_ represents the strike face area (*A*_s_ = πr^2^, *r* is the radius of the projectile); and *v* is the impact velocity. Considering the different morphologies and densities, the specific or gravimetric penetration energy is determined, which is calculated as


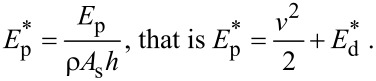


Apparently, 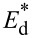 is a figure of merit to evaluate the impact energy delocalization ability of a material as more sample mass in addition to the projectile contributes to the energy dissipation.

**Figure 7:**
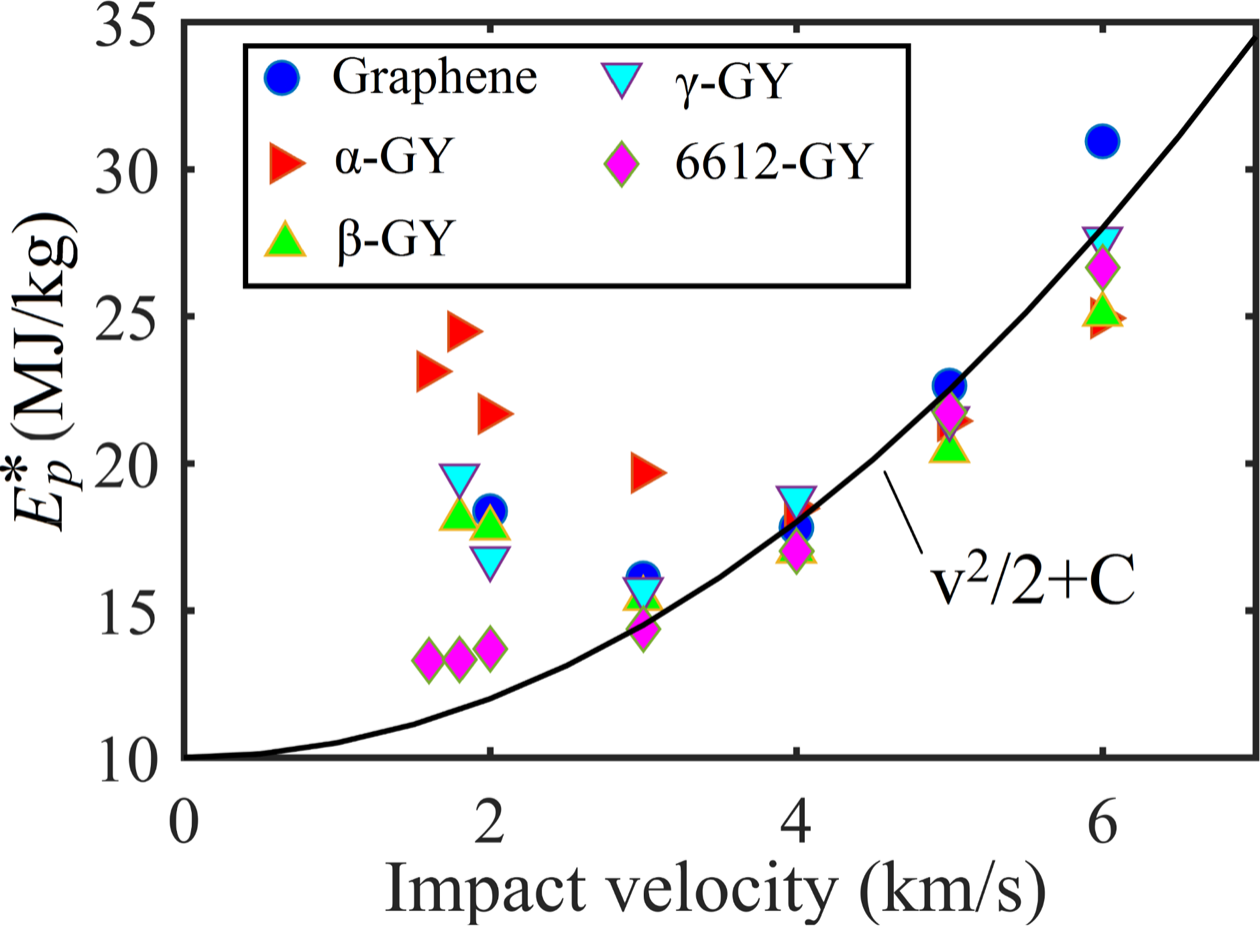
Specific penetration energy as a function of the impact velocity for GY and graphene nanosheets.

As compared in Figure 7, the specific penetration energy in each group follows a general parabolic relation for impact velocities higher than ca. 3 km/s, which is in line with the abovementioned theoretical predictions. Specifically, the 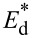 values of the GYs are found to share a shift similar to that of graphene from the material-independent energy dissipation baseline (i.e., *v*^2^/2). That is they exhibit comparable values of 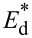. These results indicate that GYs possess a comparable energy delocalization capability to that of graphene. For impact velocities smaller than ca. 3 km/s, the penetration energy is found to deviate from the parabolic relationship with the impact velocity, which is supposed to result from the contribution of elastic deformation of the nanosheet. Particularly, α-GY is observed to possess the largest penetration energy. Recall the atomic configurations in Figure 3 and Figure 4, α-GY experiences much larger deformation (with larger cone radius and height) than the other GY counterparts and graphene. As a result, α-GY absorbs more energy at low impact velocities. However, the smaller elastic wave propagation velocity of α-GY (due to its lower Young’s modulus) will not allow it to deform sufficiently when the impact velocity increases. At an impact velocity of 6 km/s, α-GY shows the lowest penetration energy and graphene has the highest penetration energy.

In summary, the fracture behavior of monolayer GY nanosheets, namely α-GY, β-GY, γ-GY, and 6612-GY, under impact of different supersonic velocities is explored. During the deformation, crack initiates at the geometry center and the nanosheet experiences significant out-of-plane deformation before the propagation of crack. Tracking the atomic von Mises stress distribution, it is found that its cumulative density function has a strong correlation with the magnitude of the Young’s modulus of the GYs. This observation is understandable as the stress distribution is largely determined by the elastic stress propagation. For nanosheets with higher Young’s modulus, i.e., a higher elastic wave propagation velocity, it tends to transfer momentum at a faster rate. Thus, a better energy dissipation or delocalization is expected during impact. According to the simulations under impact velocity values from 1 to 6 km/s, it is observed that a higher impact velocity will induce more severe local deformation, and there will be no time for a well-developed distributed pattern as observed in scenarios with lower impact velocities. As such, extensive elastic deformation of the nanosheets will not occur under high impact velocity. Interestingly, we find that structures with a higher percentage of acetylenic linkages tend to have a lower number of breaking bonds, which means the presence of acetylenic linkages makes the structure less brittle. In particular, α-GY is observed to possess the largest penetration energy and experiences a much larger deformation (with larger cone radius and height) than the other GY counterparts and graphene at low-velocity impact. However, the relatively smaller elastic wave propagation velocity of the α-GY will not allow it to deform sufficiently when the impact velocity increases. As a result, the α-GY nanosheet absorbs more energy at low velocities up to around 4 km/s, but has the lowest energy absorption capability at higher impact velocities. This study investigates the deformation and perforation mechanism of GYs under supersonic-velocity impact for the first time. It provides insights into potential applications associated with ballistic protection in battle situations and shielding structure of spacecrafts that are exposed to space debris.

## Experimental

In the MD simulation, a low initial temperature of 10 K was chosen to minimize the influence from environmental thermal fluctuations. The widely adopted adaptive intermolecular reactive empirical bond order (AIREBO) potential was employed to describe the C–C atomic interactions within GY nanosheets [33–34], which has been shown to represent well the binding energy and elastic properties of carbonaceous materials. The C–C cut-off distance was set to be 2.0 Å to describe the C–C interaction and capture the bond breaking phenomenon during impact [2,35–36]. To differentiate the C–C atomic interactions in the GYs, Tersoff potential was chosen for the diamond projectile and a cut-off distance of 2.45 Å was selected, which has been shown to reproduce best the relation of the sp^3^-hybridized carbon atoms [37]. The interactions between the diamond projectile and the GY nanosheets were described by a Morse potential [38]. For the in silico study, the GYs were first relaxed to a minimum energy state using the conjugate gradient algorithm. Then, a Nosé–Hoover thermostat [39] was employed to equilibrate the whole system at 10 K (NVT ensemble) for 2000 fs. To capture accurately the bond breakage phenomena during supersonic-velocity impact, a time step of 0.1 fs was chosen for the simulation. The equations of motion were integrated with time using a velocity Verlet algorithm [40] with non-periodic boundary conditions applied. The thermostat was not applied during the whole process, in order to mimic kinetic and potential energy conversion accurately. To reproduce the stress distribution in each atom during the whole impact process, the atomic stress was calculated based on the virial stress Π^αβ^, which is expressed as [41]:

[1] $$\begin{array}{c} \Pi^{\alpha \beta }=\frac{1}{\Omega }\sum_{i}\omega_{i}\pi_{i}^{\alpha \beta }\;,\\ \pi_{i}^{\alpha \beta }=\frac{1}{\omega_{i}}\left(-m_{i}v_{i}^{\alpha }v_{i}^{\beta }+\frac{1}{2}\sum_{j\neq i}F_{ij}^{\alpha }r_{ij}^{\beta }\right)\;. \end{array}$$

Here, 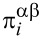 is the atomic stress associated with atom *i*. ω*_i_* is the effective volume of the *i*-th atom and Ω is the volume of the whole system. *m_i_* and *v_i_* and are the mass and the velocity of the *i*-th atom, respectively. *F_ij_* and *r_ij_* are the force and the distance between atoms *i* and *j*, respectively, and the indices α and β stand for the Cartesian components. All nanosheet samples were assumed as homogeneous continuum media with an identical thickness of *h*, which equals the thickness of single layer of graphene, i.e., 3.35 Å. Different atomic volumes inevitably alter the magnitude of the stress measurement. However, such assumption does not influence the trends of the results as focused in this paper. To visualize the stress transfer and distribution of the continuum media during the impact process, we tracked the von Mises stress σ_VM_ in the nanosheets based on the atomic virial stress (Equation 1):

[2]
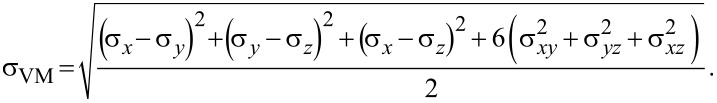


## Supporting Information

File 1 Additional experimental data.
