# Supplementary material for: Graphynes: an alternative lightweight solution for shock protection
Source: Beilstein J Nanotechnol. 2019 Jul 31;10:1588–95. doi: 10.3762/bjnano.10.154 (PMC6693407; doi:10.3762/bjnano.10.154)
Supplement: File 1 — Additional experimental data. [file Beilstein_J_Nanotechnol-10-1588-s001.pdf]

BEILSTEIN JOURNAL OF NANOTECHNOLOGY

Supporting Information

for

Graphynes: an alternative lightweight solution for shock protection

Kang Xia, Haifei Zhan, Aimin Ji, Jianli Shao, Yuantong Gu and Zhiyong Li

Beilstein J. Nanotechnol. **2019,** *10,* 1588–1595. doi:10.3762/bjnano.10.154

Additional experimental data

License and Terms: This is a supporting information file under the terms of the Creative Commons Attribution License (http://creativecommons.org/licenses/by/4.0). Please note that the reuse, redistribution and reproduction in particular requires that the authors and source are credited.
The license is subject to the *Beilstein Journal of Nanotechnology* terms and conditions: (https://www.beilstein-journals.org/bjnano)

1 Energy change of the projectile during impact

As shown in Figure S1, the kinetic energy of the project receives a significant reduction during impact, which is transferred to the nanosheet. In comparison, its potential energy shows marginal change, signifying ignorable deformation during impact. After perforation, both kinetic energy and potential energy of the project remain unchanged due to the vacuum condition.

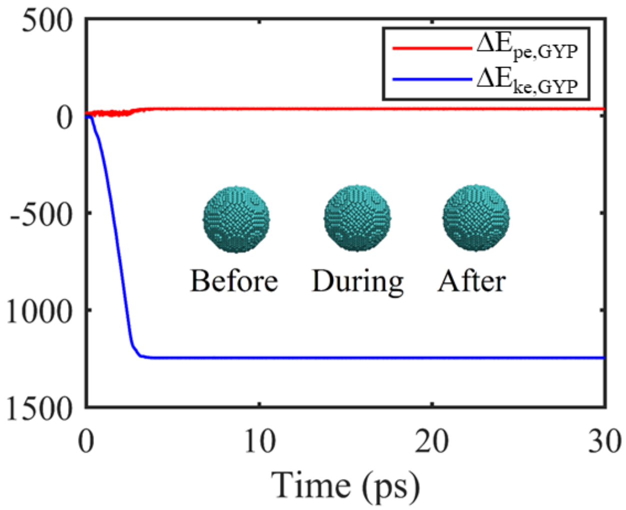

Figure S1: The kinetic energy change ($\Delta E_{\mathrm{ke,GYP}}$) and the potential energy change ($\Delta E_{\mathrm{pe,GYP}}$) of the projectile as a function of the time during impact.

2 Penetration energy of GYs under an impact velocity of 2 km/s

Comparing to α-GY, β-GY and 6612-GY are found to possess a smaller penetration energy (about 1200 eV and 1066 eV, respectively), and γ-GY exhibits a slightly higher penetration energy (ca. 1368 eV). The biggest difference of around 300 eV is found

between γ-GY and 6612-GY. Such a big difference suggests a profound impact of the morphology of the GY sheets on their impact performance.

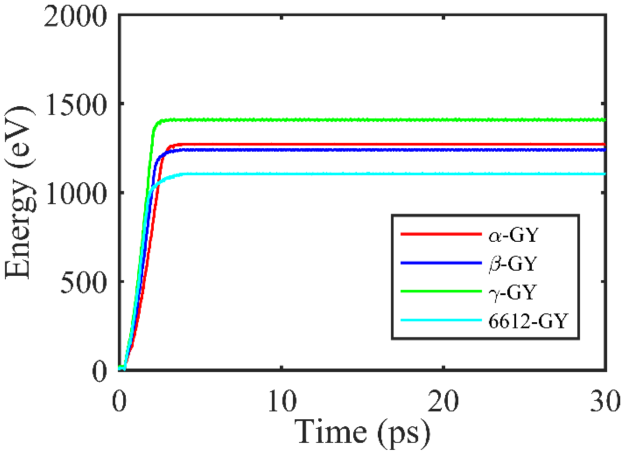

Figure S2: Energy change as a function of the time for an impact with a velocity of 2 km/s for the different GY nanosheets. The stabilized values represent the penetration energy.

3 Young's modulus of GYs and graphene

Values of the Young's modulus of the five different nanosheets are presented in Table S1. The strain rate is essential in the material characterization, thus quasi-static tensile loading is considered for characterization. The AIREBO potential is applied to describe the interaction between carbon atoms. During the simulation, the nanosheets are relaxed to a minimum energy state using the conjugate gradient algorithm. Then a Nose–Hoover thermostat [1] is applied to equilibrate the nanosheets under the NVT ensemble for 500 ps at a time step of 5 fs. The system temperature was maintained at 300 K, which is close to room temperature. For the tensile test, one end of the sample is fixed and a slow, constant velocity of 0.0005 Å/ps is applied to the other end (corresponding to a strain rate of ca. 1.25×10^{-8} ps^{-1}).

Assuming that all samples are homogenous during the simulation, the overall engineering stress is tracked, which is calculated by σ = *F*/*A*. Here, *F* is the tensile force, and *A* is the effective cross-sectional area. Specifically, the cross-sectional area is approximated as *b*·*h*, with *b* and *h* as the width and thickness of the sample, respectively. The graphite interlayer distance of 3.35 Å is taken as the thickness of the sample. Correspondingly, the engineering strain is estimated by $\varepsilon = (l - l_0)/l_0$, where *l* and l_0 are the measured and the initial length of the sample. Adopting different atomic volumes will alter the magnitude of the stress. However, the trends of the results in this paper will not change. For comparison, the effective Young's modulus is extracted from the stress–strain curve by linear fitting. The atomic stress is calculated based on the virial stress, which is expressed as [2]:

$$\Pi^{\alpha\beta} = \frac{1}{\Omega}\sum_i \varpi_i \pi_i^{\alpha\beta}, \quad \pi_i^{\alpha\beta} = \frac{1}{\varpi_i}\left(-m_i v_i^{\alpha} v_i^{\beta} + \frac{1}{2}\sum_{j\neq i} F_{ij}^{\alpha} r_{ij}^{\beta}\right) \tag{S1}$$

Here, $\pi_i^{\alpha\beta}$ is the atomic stress associated with atom *i*. ω_i is the effective volume of the *i*-th atom and Ω is the volume of the whole system. m_i and v_i and are the mass and the velocity of the *i*-th atom, respectively. F_{ij} and r_{ij} are the force and the distance between atoms *i* and *j*, respectively, and the indices α and β stand for the Cartesian components. In terms of the magnitude of Young's modulus, our results show good agreement with published results [3,4] and demonstrate a correlation between the magnitude of Young's modulus and the CDF profile.

Table S1: Young's modulus (TPa) of different GYs and graphene. *x*- and *y*-axis are the lattice directions shown in Figure 1. For graphene and α-GY, *x*- and *y*-axis are the zigzag and armchair lattice directions, respectively. The values in brackets are from theoretical calculations based on the reported Young's modulus from literature [3,4].

lattice direction	graphene	α-GY	β-GY	γ-GY	6612-GY
x-axis	0.981 (0.995)	0.131 (0.12)	0.272 (0.261)	0.504 (0.505)	0.441 (0.445)
y-axis	0.985 (0.996)	0.132 (0.119)	0.286 (0.260)	0.505 (0.508)	0.33 (0.35)

4 Deformation of GYs under an impact velocity of 6 km/s

To simulate the behaviour of GYs under high velocity impact, we compared the atomic configurations of GYs with projectile velocity of 60 Å/ps. The impact area melted immediately when the projectile approached the GY nanosheets, which created a large number of dangling bonds for both cases presented (insert of Figure S3a and Figure S3c). In addition to that, a clear tensile stress wave surrounds the impact area is found, which spreads quickly to rest of the sample (Figure S3b and Figure S3d).

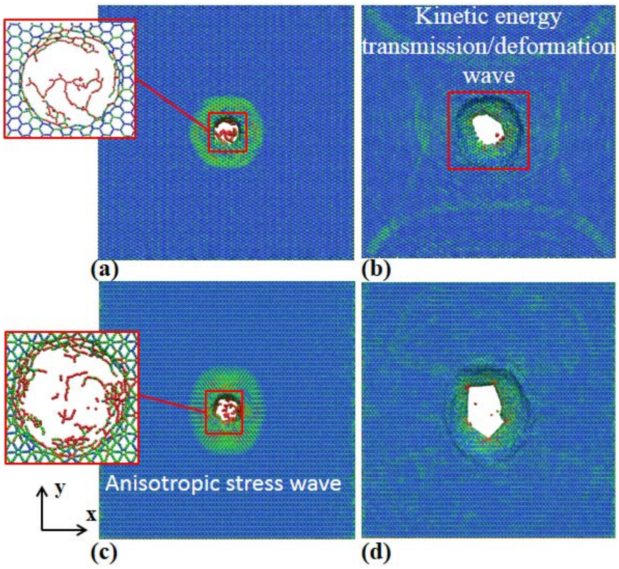

Figure S3: Stress distribution for α-GY nanosheet under projectile velocity of 6 km/s at: (b) 0.6 ps (c) 2.5 ps. The inserts schematically show the atomic configuration of the final failure shape of the nanosheet; stress distribution for 6612-GY nanosheet under projectile velocity of 6 km/s at (d) 0.6 ps (e) 4.2 ps.

5 Number of breaking bonds of GY and graphene nanosheets (time step 0.5 fs)

Additionally, we also examined the results by varying the time step from 0.1 to 0.5 fs. Figure S4 shows the number of breaking bonds of GY and graphene nanosheets under different impact velocity amplitude using time step 0.5 fs. For each case, similar results are obtained using a time step of 0.1 fs (Figure 6)

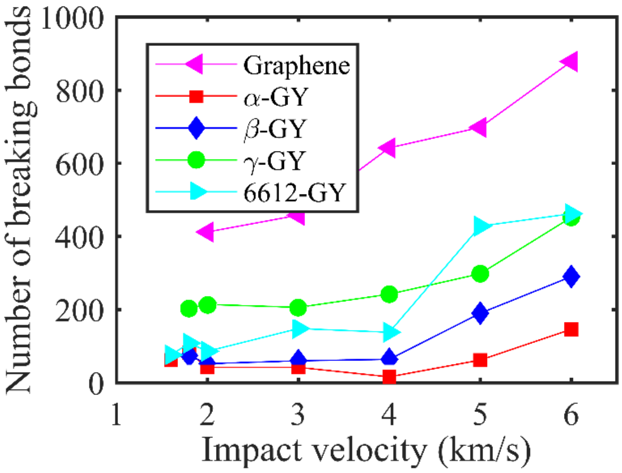

Figure S4: Number of breaking bonds of GY and graphene nanosheets under different impact velocity amplitude using time step 0.5 fs.

References

1. Hoover, W. G. *Phys. Rev. A* **1985,** *31,* 1695. doi:10.1103/PhysRevA.31.1695
2. Diao, J.; Gall, K.; Dunn, M. L. *J. Mech. Phys. Solids* **2004,** *52,* 1935–1962. doi:10.1016/j.jmps.2004.03.009
3. Zhang, Y.; Pei, Q.; Wang, C. *Appl. Phys. Lett.* **2012,** *101,* 081909. doi:10.1063/1.4747719
4. Hernandez, S. A.; Fonseca, A. F. *Diamond Relat. Mater.* **2017,** *77,* 57–64. doi:10.1016/j.diamond.2017.06.002
